# Smad4-expression is decreased in breast cancer tissues: a retrospective study

**DOI:** 10.1186/1471-2407-6-25

**Published:** 2006-01-26

**Authors:** Christina H Stuelten, Miriam B Buck, Juergen Dippon, Anita B Roberts, Peter Fritz, Cornelius Knabbe

**Affiliations:** 1 Department of Clinical Chemistry, Robert Bosch Hospital, Stuttgart, Germany; 2 Dr. Margarete Fischer-Bosch Institute of Clinical Pharmacology, Stuttgart, Germany; 3 University of Stuttgart, Department of Mathematics, Stuttgart, Germany; 4 NIH, NCI, Laboratory of Cell Regulation and Carcinogenesis, Bethesda, MD, USA

## Abstract

**Background:**

Although transforming growth factor β (TGF-β) typically inhibits proliferation of epithelial cells, consistent with a tumor suppressor activity, it paradoxically also exhibits pro-metastatic activity in the later stages of carcinogenesis. Since tumors often display altered TGF-β signaling, particularly involving the Smad-pathway, we investigated the role of Smad4-expression in breast cancer.

**Methods:**

Smad4 expression was investigated by immunohistochemistry in formalin-fixed, paraffin-embedded tissue from 197 samples of primary breast cancer obtained between 1986 and 1998. The prognostic value of Smad4-expression was analyzed.

**Results:**

Smad4 expression was found to be reduced in lobular and ductal breast carcinoma as compared to surrounding uninvolved lobular and ductal breast epithelia (p < 0.001, n = 50). Smad4-expression correlated positively with expression of TGF-β-receptor I (p < 0.001, n = 197) and TGF-β-receptor II (p < 0.001, n = 197), but showed no significant correlation with tumor size, metastases, nodal status, histological grade, histological type, or estrogen receptor expression. While not achieving statistical significance, there was a trend towards longer survival times in patients with Smad4 negative tumors.

**Conclusion:**

According to the suggested role of Smad4 as a tumor suppressor we observed that expression of Smad4 is lower in human breast cancer than in surrounding breast epithelium. However, we also observed a trend towards longer survival times in Smad4-negative patients, indicating the complex role of TGF-β signaling in tumor progression.

## Background

Transforming growth factor beta (TGF-β) is an important regulator of epithelial cell growth. Conflicting data exist about the influence of TGF-β on the development and progression of breast cancer. The growth of many human breast cancer cell lines is inhibited by TGF-β [1,2] due to an inhibition of cell division and an induction of apoptosis. This is consistent with a tumor suppressor effect in well-differentiated tumors [3,4]. On the other hand, certain highly aggressive breast cancer cell lines are refractory to suppressive effects of TGF-β on cell growth and may acquire sensitivity to pro-metastatic effects of TGF-β in later stages of tumorigenesis [5-8].

Smad proteins are the principal transducers of signals from TGF-β. TGF-β binds to homodimers of the TGF-β type II receptor (TβRII) which recruits and activates homodimers of TGF-β type I receptor (TβRI) serine/threonine kinase. Activated TβRI phosphorylates Smad2 or Smad3 [9,10] which heterodimerize with Smad4. These heterocomplexes translocate into the nucleus where they bind DNA and regulate TGF-β dependent gene expression [11]. TGF-β signaling is altered in various tumors. We have recently shown that loss of TβRII expression correlated significantly with better prognosis in estrogen receptor negative breast cancer patients [12], but little is known about the influence of the various downstream TGF-β signal transduction pathways on breast cancer prognosis. Deletions or mutations of Smad4 (also known as DPC4, deleted in pancreatic cancer), a tumor suppressor [13] and the only known co-Smad protein in TGF-β-, activin- and bone morphogenetic protein-signaling, are found in various epithelial tumors, including pancreatic carcinoma [14,15], esophageal carcinoma [16], colorectal carcinoma [17], renal cell carcinoma [18], as well as breast carcinoma [19,20]. However, little is known about the expression level of Smad4 or its prognostic significance in breast cancer.

In order to gain further insight into the status of Smad signal transduction in breast cancer progression, we used immunohistochemistry to analyze the expression of Smad4 in archival tissues of 197 breast cancer patients. Smad4 expression in tumor tissues was compared to that of surrounding normal tissues and correlated to established prognostic markers for breast cancer as well as overall survival.

## Methods

### Specimens

Formalin-fixed, paraffin-embedded invasive breast carcinomas collected from 1987 to 1998 were retrieved from the archives of pathology of the Robert Bosch Hospital Stuttgart. Clinical and pathological data were retrieved from clinical databases as well as from the archives of pathology and included estrogen receptor, progesterone receptor, TGF-β-receptor I, TGF-β-receptor II, tumor size, nodal metastases, metastases, histological grade, tumor stage, histological type, age, menopausal status, therapy, and survival time of patients. Cases with incomplete clinical data sets or without sufficient archived tissues were excluded from the study (number of included cases: 197, number of excluded cases: 69, drop out rate: 25.9 %).

### Cell cultures

MCF-7 and MDA468 cells were grown in Dulbecco's modified Eagles medium (DMEM, Invitrogen, Carlsbad, USA) supplemented with 10% fetal calf serum (Invitrogen). Culture of MCF10CA1a, MCF10CA1h, MCF10A1k.cl2, and MCF10A cells is described elsewhere [21].

### Detection of Smad4

3 μm paraffin sections were dewaxed by routine techniques. Immunohistochemistry was performed by the EnVision-technique (DAKO, Hamburg, Germany) using an Autostainer (DAKO). In brief, slides were soaked with antigen retrieval buffer (DAKO) and microwave-treated (3 × 5 min, 600 W) followed by treatment with blocking solution (methanol/H_2_O_2_) to block endogenous peroxidase. Slides were incubated at room temperature for 30 min with a monoclonal antibody to Smad4 (clone B-8, Santa Cruz Biotechnology Inc, Heidelberg, Germany) diluted 1:50 with antibody dilution medium (DAKO), and for 30 min with peroxidase-labeled polymer (DAKO). Anti-Smad4-antibody was detected by 3,3'-diaminobenzidine/ H_2_O_2 _containing staining solution. Sections were counterstained with hematoxylin. Negative controls were set up by omitting the primary antibody or the peroxidase labeled polymer. As a positive control specimens were stained with a different primary antibody to Smad4 (clone DCS-46, acris, Bad Nauheim, Germany) which yielded results identical to clone B-8 (data not shown). Immunostaining using an antibody to cytokeratin 18 (DAKO) was used as an additional control for the staining method.

To obtain specimens for immunocytochemistry, cells were grown on sterile coverslips to 70% to 80% confluence and fixed in acetone (-20°C, 5 min). For detection of Smad4 the protocol described above was slightly modified: No antigen retrieval was performed and specimens were incubated with the primary antibody (anti-Smad4, 1:50 in TBST) at room temperature overnight.

Immunohistological labeling was evaluated for tumor tissues as well as for adjacent, unaffected breast epithelia (i.e. breast epithelia not containing DCIS, LCIS or atypical ductal hyperplasia) by scoring staining intensity as: 0 (negative), 1 (weak), 2 (moderate), 3 (strong) as well as by scoring the fraction of Smad4-positive tumors cells: 1 (0% to 20%), 2 (21% to 50%), 3 (51% to 80%), 4 (81% to 100%). An Immunoreactive Score (IRS) was calculated by multiplying "staining intensity score" by "fraction of positive cells"[22]. IRS = 0 was defined as Smad4 – negative, an IRS between 1 and 12 (1≤IRS≤12) as Smad4-positive. Specimens were scored by 2 investigators and in cases of conflict, specimens were re-evaluated together.

### Detection of TGF-β receptors

Immunostaining of TβRI and TβRII was described earlier [12].

### Immunoblotting

For Western blots cells were grown to 80% – 90% subconfluent monolayers and processed as described elsewhere [23] using a monoclonal antibody against Smad4 (clone B-8, Santa Cruz Biotechnology Inc, Santa Cruz, California, USA), or a monoclonal antibody against α-tubulin (Zymed Laboratories Inc, San Francisco, California, USA).

For dot blots tumor tissues were snap frozen, homogenized by use of a Mikro Dissmembrator S (Braun Biotech International, Melsungen Germany), and incubated with lysis buffer (50 mM Tris/HCl pH7.6, 250 mM NaCl, 5 mM EDTA, 0.1% (v/v) Triton X-100) supplemented with the protease inhibitor complete (Roche Applied Science, Mannheim, Germany). Insoluble components were pelleted (12000 rpm, 4°C, 15 min) and protein concentrations were determined by the Bradford protein assay (Bio-Rad Laboratories GmbH, Munich, Germany). Samples were dotted onto a nitrocellulose membrane (Schleicher&Schuell, Dassel, Germany). Dot blots were probed with anti Smad4 – antibody (clone B-8, Santa Cruz Biotechnology Inc), detected with alkaline phosphatase-conjugated secondary antibody (DAKO, Hamburg, Germany) and developed using BCIP/NBT (Roche Applied Science) solution (100 mM Tris/HCl pH9.5, 100 mM NaCl, 50 mM MgCl_2_, NBT 100 μl/100 ml, BCIP 75 μl/100 ml)

### Statistical analysis

IRS values calculated for breast cancer and adjacent normal breast epithelia originating from the same slide were compared by paired two-sample sign test. Cross tabulations were analyzed by Spearman's rank correlation test. Survival time analysis was done by the Kaplan-Meier estimator [24] and the log-rank test [25]. p-values < 0.05 were considered to be significant. No p-value was adjusted for possible multiple testing. Analysis was performed using SPSS version 10.0 for both, patient with "unknown cause of death" classified as "death due to cancer" and as "death not due to cancer", and similar results were obtained for both analyses; results shown in this work were obtained by the analyses classifying "unknown cause of death" as "death not due to cancer".

## Results

### Patients

The median age of the study population was 56 years (range: 26 years to 86 years). The median follow-up was 68 months (range 2 months to 184 months). 96 % of patients had stage I, II or III breast cancer (stage I: 17.7 %, stage II: 54.8%, stage III: 23.9 %, stage IV: 3.6 %). After surgery was performed 103 patients received radiotherapy, and 158 patients underwent chemotherapy. Further characteristics (TNM staging, histological grade, histological type, steroid receptor status, and TGF-β-receptor status) are listed in Table 1. Survival time analysis demonstrated that well known prognostic factors significantly influenced 5-year survival time in our collective (tumor size, T, p = 0.0002; nodal status, N, p = 0.0002; metastases, M, p < 0.001; WHO stage, p < 0.001).

### Immunostaining of Smad4 can be used to determine Smad4 expression

**Figure 1:**
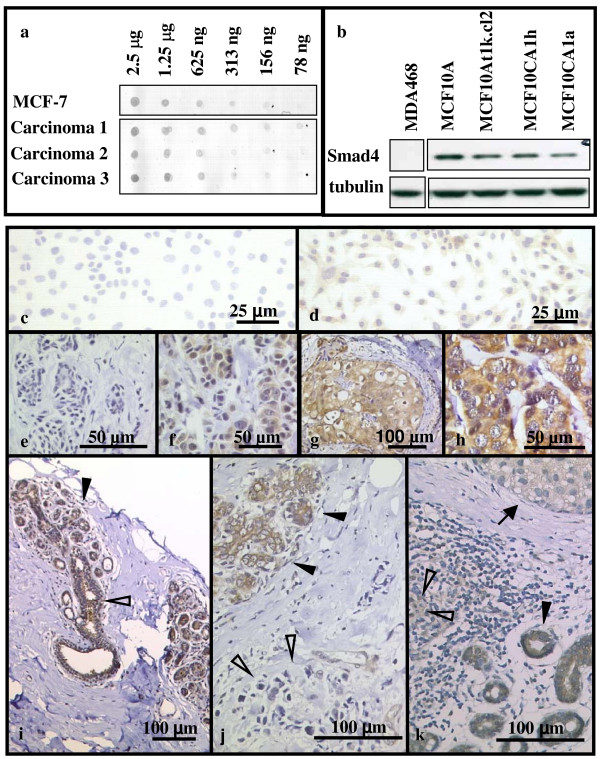
**Detection of Smad4 in breast cancer cell lines, tumor tissues and surrounding normal breast epithelia using the monoclonal antibody clone B-8**. (a) Detection of Smad4 in dilution series of total protein lysates of breast cancer tissue and MCF7 cells by dot blot. (b) Detection of Smad4 expression in Smad4 negative MDA468 cells, non malignant, immortal MCF10A cells, and Ras-transformed, increasingly malignant MCF10A derived cell lines by Western blot. Immunohistochemical detection of Smad4 in breast cancer cell lines (c: MDA 468; d: MCF10CA1a), breast carcinoma (e-h, j,k), and surrounding normal breast epithelia (i). Staining intensity varied between specimens: none (c,e), weak (f), moderate (d,g), and strong (h). Cytoplasm as well as nuclei stained positive (d,f-h). Within the same specimen tumor cells stained less than surrounding normal epithelia. (i) Lobular (closed arrowhead) and ductal (open arrowhead) breast epithelium, (j) lobular breast epithelium (closed arrowhead) and lobular breast carcinoma (open arrowhead), (k) ductal breast epithelium (closed arrowhead), ductal breast carcinoma (open arrowhead) and ductal breast carcinoma *in situ *(arrow).

Western blotting of lysates of human breast cancer cell lines revealed that the monoclonal antibody to Smad4 correctly identified a protein of 63kDa, just slightly smaller than ectopically expressed flag-tagged human Smad4 used as a positive control (data not shown). A dot blot set up with serial dilutions of total protein extracts from tumor tissues as well as MCF-7 cells showed decreasing signal intensities with decreasing Smad4-concentrations (Fig. 1a). Western Blot analysis of breast cancer cells of increasing malignancy (MCF10A, MCF10At1k.cl2, MCF10CA1h, MCF10CA1a [26-28]; Fig. 1b) showed that Smad4 expression decreased with increasing malignancy of the tumor cell line (Fig. 1b). MDA-MB-468 cells, which do not express Smad4 due to a deletion of the Smad4 gene [29], served as negative control (Fig. 1b). Consistently, immunostaining was positive in MCF10CA1a cells whereas MDA-MB-468 cells remained Smad4 negative (Fig. 1c,d). These results demonstrated that anti-Smad4 clone B-8 could be used to determine Smad4 expression in breast cancer tissues by immunohistochemistry, as already described by other authors [14,15].

### Smad4 expression in tumor tissues and surrounding normal tissues

**Figure 2:**
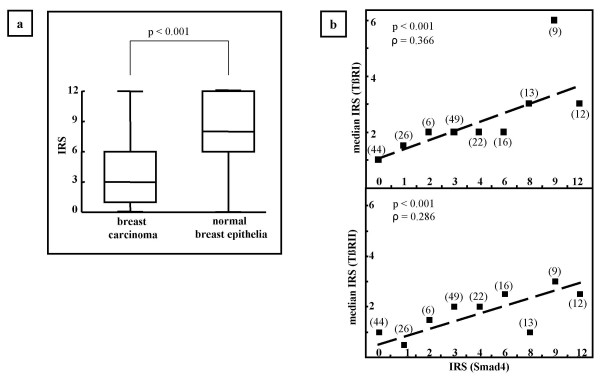
(a) Expression of Smad4 as indicated by IRS in lobular and ductal breast carcinoma compared to surrounding normal lobular and ductal breast epithelia of the same specimen (n = 50). Median, 1. and 3. quartile, highest and lowest IRS-value are shown (p < 0.001, sign-test). (b) Correlation according to Spearman between expression of Smad 4 and expression of TβRI (upper panel) and TβRII (lower panel), respectively (n = 197). For each IRS value of Smad4 the median IRS of TβRI and TβRII is denoted by a square. For exploratory reasons a regression line is fitted.

To investigate whether this same inverse relationship of Smad4 staining and degree of malignancy observed in the MCF10A-derived series of cell lines would be seen in human breast cancers, we performed an immunohistochemical analysis of the expression of Smad4 in archival tissues of 197 breast cancer patients. Immunohistochemistry revealed both cytoplasmic and nuclear staining for Smad4 in both normal breast epithelia and breast carcinomas (Fig. 1e–k). There was no significant change in the intracellular localization of Smad4 in the tumor tissues as compared to the surrounding normal epithelium (data not shown). The staining intensity varied greatly in both uninvolved breast epithelia and tumor tissues from different patients (Fig. 1e–h). Applying the IRS criteria (see Materials and Methods), 23% of the tumor tissues were Smad4-negative (IRS = 0), and a further 41 % stained weakly positive (1 ≤ IRS ≤ 3). In general, Smad4 immunostaining was stronger in the surrounding normal breast epithelia than in tumor tissues. Therefore Smad4 expression of tumor tissues and the surrounding normal tissues was compared (Fig. 1i–k). Overall Smad4-expression was significantly lower in breast carcinoma (median IRS: 3) than in normal breast epithelia (median IRS: 8, p < 0.001, n = 50, sign test; Fig. 2a). This effect was robust when subgroups were formed (lobular carcinoma, ductal carcinoma): Lobular breast epithelia had a median IRS of 12, while the median IRS of lobular carcinomas was 2.5 (p = 0.031, n = 6, sign test). Ductal breast epithelia had a median IRS of 8 whereas ductal breast carcinomas had a median IRS of 3 (p < 0.001, n = 44, sign test). Thus these data are consistent with the pattern observed in the series of cell lines using Western blotting where Smad4 expression was higher in the less malignant MCF10A and MCF10At1k.cl2 cells as compared to the xenograft forming cell lines MCF10CA1h and MCF10CA1a (Fig. 1b).

### Correlation of the expression of Smad4 and TGF-β-receptors

Since Smad4 is a key component of the TGF-β-signaling pathway, we investigated its expression compared to that of the TGF-β-receptors. Smad4-expression correlated significantly with the expression of TβRI (ρ_S _= 0.366, p < 0.001, Spearman's rank correlation test; Fig. 2b, upper panel) and TβRII (ρ_S _= 0.286, p < 0.001, Spearman's rank correlation test; Fig. 2b, lower panel) as well as with the coexpression of TβRI and TβRII (ρ_S _= 0.345, p < 0.001, Spearman's rank correlation test, data not shown).

### Correlation of Smad4-expression with prognostic parameters and influence of Smad4 on overall survival time

Further analysis of the data revealed no significant correlation between Smad4-expression (negative: IRS = 0, positive: 1≤IRS≤12) and other established prognostic parameters (tumor size, nodal status, metastases, histological grade, histological type, WHO stage, and steroid receptors, Table 1). While not achieving significance, the survival curve of Smad4-negative patients (5-year survival rate: 92.4%) was slightly better than that of Smad4-positive patients (5-year survival rate: 74.5%, median follow-up time: 68 months, p = 0.187, log-rank test; Table 2). Stratifying the collective with regard to the WHO stage not only showed a trend for longer survival in the group of Smad4 negative patients compared to Smad4 positive patients, but also revealed that this effect became most prominent, though still not statistically significant, at WHO stage III patients that already have positive lymph nodes but no distant metastasis (Table 2). In our collective, the group WHO stage IV included only 7 patients, 1 of them being Smad4 negative, so that results of the statistical analysis of this group were not meaningful. However, pooling of the groups WHO stage I and II as well as WHO stage III and IV again showed a trend toward a positive influence of Smad4 loss on 5-year survival that was pronounced in advanced tumor stages (Table 2). For further analysis, we stratified our dataset with regard to tumor size, nodal status (Table 2) and distant metastasis (data not shown). Overall, loss of Smad4 expression correlated with increased 5-year survival rates in these groups, but again without reaching statistical significance. The most distinct, but not statistically significant, impact of Smad4 loss was seen as a trend for an increase in 5-year survival from 59% to 91.1% in the group N1.

Because of the central role of Smad4 in TGF-β signaling and the well-described cross-talk of TGF-β and estrogen signaling [30-32], survival curve analysis was performed after stratifying the collective with regard to ER, TβRI, and TβRII (Table 2). Although no significant influence of Smad4 expression on survival curves could be shown for any group, nevertheless there was a trend towards higher 5-year survival rate in TβRII negative, Smad4 negative patients (5-year survival rate: 100%, n = 19) than in TβRII negative, Smad4 positive patients (5-year survival rate: 80%, n = 39), or TβRII positive, Smad4 negative patients (5-year survival rate: 85%, n = 25,) and TβRII positive, Smad4 positive patients (5-year survival rate: 72.8%, n = 114). Taken together, patients with a "double hit" in the TGF-β pathway had the highest 5-year survival rate, followed by patients with a "single hit", whereas those with intact signaling showed the worst survival. There was no survival effect for the presence or absence of TβRI, which is much less prevalently altered in cancer than the ligand-binding TβRII, whereas the effect of Smad4 could still be seen in this stratification. Stratifying for ER again revealed a trend for longer 5-year survival rate in Smad4 negative patients than in Smad4 positive patients, as well as a trend towards a longer 5 year survival rate in Smad4 positive, ER positive patients (78.9%) as compared to Smad4 positive, ER negative patients (66.5%; Table 2), consistent with the known effect of the ER status on survival.

## Discussion

We have used immunohistochemical staining to show that Smad4 expression is markedly decreased in breast cancer compared to surrounding normal breast epithelium. Smad4 staining correlated with the expression of TβRI and TβRII. In Smad4 negative patients of stage III breast cancer, i.e. in patients with positive lymph nodes at primary diagnosis, a trend for an increased 5-year survival was observed.

Although the expression of Smad4 in breast cancer tissues as well as in normal epithelia varied greatly between specimens from individual patients, Smad4 expression was significantly reduced in tumor tissues as compared to the surrounding normal epithelia within the same specimen. Overall, we found that 23% of the specimens were Smad4 negative and that a further 41% stained weakly. In contrast, a tissue microarray analysis of Smad4 expression in 456 cases of breast carcinomas by Xie et al. [33], reported that only 2% breast cancer tissues were Smad4-negative. This difference might be due to the different age of the specimens, which was up to 70 years in the set used by Xie et al. [33], and to staining methods employing different secondary detection methods, as well as to the different analysis of the specimens. Another recently published paper [34] showed that Smad4 mRNA expression is reduced in ductal carcinoma as compared to normal tissues. At the protein level, we have shown that in the MCF10-system of genetically related cell lines of differing degrees of malignancy, Smad4 levels were higher in non-malignant MCF10A cells than in the increasingly malignant cell lines MCF10At1k.cl2, MCF10CA1h, and MCF10CA1a, indicating that decreased Smad4 protein expression might accompany tumor progression from early stages on *in-situ *and *in-vivo*. The reduced expression of Smad4 observed in this study could have resulted either from decreased transcription rates from the *Smad4 *gene or from increased degradation of the Smad4 protein. The latter appears more probable as various studies have shown that the steady state level of Smad4 is tightly regulated by ubiquitinylation [35,36] and sumoylation [37,38].

TGF-β inhibits cell proliferation in breast epithelial cells and many breast cancer cell lines [21]. In our specimens, Smad4 expression in tumor tissue was lower than in surrounding epithelia, indicating impaired TGF-β signaling and possible escape from TGF-β-dependent growth inhibition. Given the central role of Smad4 in the signaling of all TGF-β-related superfamily members, reduced or absent Smad4-expression as described here would be expected to alter the signaling not only of TGF-β, but also of the BMPs and activins, which also can inhibit proliferation of breast cancer cells [39-41]. Of these proteins, TGF-β, that additionally signals via MAPK-pathways [42] and interacts with ER signaling [30], paradoxically also exhibits pro-metastatic activity in later stages of cancer progression [7,42]. Consistent with this pro-metastatic role of TGF-β we have previously shown that loss of TβRII expression correlated significantly with better prognosis in estrogen receptor negative breast cancer patients [12]. In this work we show that loss of Smad4 expression as a trend correlates with increased survival times. Although not reaching significance, this effect was found in all subgroups. Recent data showing that growth of primary xenografted tumors of MDA-MB-231 human breast cancer cells in mice is not affected by the Smad4 status, whereas metastasis of these cells to bone is Smad4-dependent, provide a basis for the trend we have observed of increased survival times in Smad4 negative patients[43]. This trend showing an effect of Smad4 on survival time was most prominent in TβRII negative patients indicating that silencing of TGF-β signal transduction via the Smad pathway might improve patient survival. Similarly, the trend towards higher 5-year survival rate of Smad4 positive, ER positive patients as compared to Smad4 positive, ER negative patients might be due to interaction of ER and Smad3 that is known to inhibit TGF-β signal transduction [30], again presumably reducing the pro-metastatic effect of TGF-β.

Survival in breast cancer patients that undergo surgery of the primary tumor greatly depends on the systemic spread of the tumor and complications caused by metastasis. In our collective, loss of Smad4 showed a trend towards improved survival of patients with advanced disease (WHO stage 3/4), and was particularly striking for WHO stage 3, whereas the data of patients at stage 4 were weak due to the low case number. This effect was likely due to the benefit of loss of Smad4 in patients with positive nodal status, as Smad4 negative patients staged N1 had a 50% increased 5-year survival rate as compared to Smad4 positive patients. Similarly, biallelic loss of TβRII expression due to microsatellite instability has previously been reported to positively influence the prognosis particularly of node positive patients suffering from colon cancer [44].

In our studies loss of Smad4 and TGFβRII showed a trend for longer survival times in subgroups (N1 or ER negative, respectively), but loss of Smad2-phosphorylation was previously correlated with a worse all-over survival in node positive breast cancer [33]. On the other hand, in-vivo data obtained by employing different mouse models of breast cancer [8,21,23,45] indicate that reducing TGF-β-signaling by impairing TβRII, TβRI kinase activity or Smad3-phosphorylation enhances development of the primary lesion but reduces metastasis whereas constitutive activation of TβRI has opposite effects. These seemingly contradictory results might be due, at least in part, to the distinct roles of Smad2, Smad3, and Smad4 in TGF-β signaling in the changing signaling context of cancer progression, resulting in altered target gene expression and ultimately different biological effects. TGF-β signaling via canonical and non-canonical pathways is complex, and the all-over effect of TGF-β on cell behavior is context dependent. Based on this complexity, the power of the exploratory study presented here is limited, although the results are consistent with the current literature. It will be the goal of future work to further elucidate the role of TGF-β and the proteins involved in TGF-β signal transduction in progression of breast cancer.

## Conclusion

Our data show that Smad4 expression in breast cancer is lower than in normal adjacent breast epithelial tissue and imply that impairment of TGF-β/Smad-signaling because of loss of TβRII or Smad4 might improve 5-year survival by possibly slowing down metastases. Our data from human tumors, although not achieving statistical significance, are in agreement with results obtained in vitro and in animal models, where reduced TGF-β signaling provides a survival benefit in advanced tumor stages due to decreased metastases.

## List of abbreviations

BMP bone morphogenetic protein, ER estrogen receptor, MAPK mitogen activated protein kinase, TβRI TGF-β receptor 1, TβRII TGF-β receptor II, TGF-β transforming growth factor β

## Competing interests

The author(s) declare that they have no competing interests.

## Authors' contributions

CHS, MBB, ABR, PF, and CK were responsible for generating the hypothesis and correcting the manuscript. PF was responsible for collecting the patient material. CHS and PF were responsible for Smad 4 immunostaining, examination and interpretation of the results. MBB and PF contributed the TβRI and TβRII datasets. CHS, PF and JD performed the statistical analysis of the data. CHS, ABR, PF and CK were responsible for writing the manuscript. All authors read and approved the final version of the manuscript.

## Pre-publication history

The pre-publication history for this paper can be accessed here:



## Figures and Tables

**Table 1:** Association of Smad4-expression and breast cancer characteristics (n = 197). In case of histological type carcinoma other than ductal invasive or lobular invasive were excluded from statistical analysis (resulting n = 178). Data were calculated by Spearman's rank correlation test.

	Smad4 negative	Smad4 positive	rho_s _(95% confidence interval)	p
tumor size				
T_1_	15	37	0.007	0.924
T_2_	15	84	(-0.133, 0.147)	
T_3_	8	12		
T_4_	6	20		
nodal status				
N_0_	18	64	-0.002	0.977
N_1_	23	77	(-0.142, 0.138)	
N_2_	2	8		
N_3_	1	4		
metastases				
M_0_	43	147	0.037	0.605
M_1_	1	6	(-0.102, 0.176)	
histologic grade				
G_1_	3	4	0.004	0.956
G_2_	25	97	(-0.136, 0.144)	
G_3_	16	52		
histological type				
ductal carcinoma	29	127	-0.144	0.055
lobular carcinoma	8	14	(-0.285, 0.003)	
(others)	7	12		
WHO stage				
I	11	24	0.22	0.760
II	19	89	(-0.118, 0.161)	
III	13	34		
IV	1	6		
ER				
positive	28	94	0.019	0.793
negative	16	59	(-0.122, 0.157)	
PR				
positive	30	90	0.08	0.265
negative	14	63	(-0.061, 0.218)	
TβRI				
positive	27	129	0.235	**0.001**
negative	17	24	(0.098, 0.532)	
TβRII				**0.023**
positive	25	114	0.162	
negative	19	39	(0.022, 0.295)	

**Table 2:** Analysis of survival time of Smad4-positive and Smad4-negative patients stratified with regard to tumor stage, ER-status, TβRI-status, and TβRII-status. 5-year survival rates were computed by the Kaplan-Meier estimator. Survival curves were compared by log-rank test (n = 197).

	5 year survival		Total number		Log rank test		
					
					Number censored		p
	Smad4 negative	Smad4 positive	Smad4 negative	Smad4 positive	Smad4 negative	Smad4 positive	

Unstratified	92.4	74.5	44	153	35	111	0.187
WHO I	100.0*	86.7	11	24	11	19	0.135
WHO II	94.4	81.3	19	89	15	71	0.821
WHO III	**92.3**	**54.7**	13	34	9	19	0.137
WHO IV	0	20.8	1	6	0	2	0.661
WHO I/II	96.2	82.5	30	113	26	90	0.32
WHO III/IV	**84.4**	**50.2**	14	40	9	21	0.121
T_1_	100.0	83.7	15	37	14	29	0.209
T_2_	93.3	71.2	15	84	11	57	0.448
T_3_	87.5	63.5	8	12	5	8	0.899
T_4_	50.0	45.5	6	20	2	9	0.588
N_0_	94.1	88.1	18	64	16	52	0.425
N_1_	**91.1**	**59.0**	23	77	15	45	0.167
N_2_	50.0	52.5	2	8	1	4	0.666
N_3_	0	50.0	1	4	0	2	0.948
N_1–3_	**84.3**	**57.9**	26	89	16	51	0.227
ER negative	93.8	66.5	16	59	13	41	0.278
ER positive	91.7	78.9	28	94	22	70	0.474
TβRI negative	94.1	76.5	17	24	16	19	0.245
TβRI positive	92.3	74.1	27	129	19	92	0.523
TβRII negative	**100.0**	80	19	39	17	30	0.133
TβRII positive	85.9	72.8	25	114	18	81	0.806

* survival times could not be computed as all cases were censored
